# Case Report: A rare case of mixed epithelial and stromal tumor of the kidney in an adolescent: imaging findings and literature review

**DOI:** 10.3389/fped.2025.1550425

**Published:** 2025-05-08

**Authors:** Guang Fu, Rong Xiao, Xudan Yang, Tao Lu

**Affiliations:** ^1^ Department of Radiology, Sichuan Provincial People’s Hospital, University of Electronic Science and Technology of China, Chengdu, China; ^2^ Department of Pathology, Sichuan Provincial People’s Hospital, University of Electronic Science and Technology of China, Chengdu, China

**Keywords:** mixed epithelial and stromal tumor of the kidney, kidney, adolescent, magnetic resonance imaging, computed tomography

## Abstract

Mixed epithelial and stromal tumor of the kidney (MESTK) is an unusual biphasic benign renal neoplasm. It predominantly occurs in perimenopausal women, with only eight cases reported in children. Owing to its rarity, only limited radiological information has been reported in the literature, in none of the previous pediatric cases were both CT and MRI findings of the tumor provided. Herein, we report a rare case of MESTK in a 13-year-old girl. This case was the largest observed in children to date. Findings from our report provide novel insights into the MRI features of a large pediatric MESTK and indicate the importance of MRI for observing adipose components and the absence of diffusion restriction within the tumor. Radiologists should consider the possibility of MESTK in children when they observe a large, solid renal tumor without diffusion restriction in children.

## Introduction

Mixed epithelial and stromal tumor of the kidney (MESTK) is an unusual benign renal neoplasm. It is characterized by a mixture of stromal and epithelial structures, and it accounts for 0.2%−0.28% of all renal neoplasms (1–3). The age of affected patients ranges from 18 to 82 years, with a female preponderance (approximately 1:10 male-to-female ratio) (4–6). Most MESTKs occur in perimenopausal females (age: 40–50 years), and the pathogenesis of the tumor is related to long-term estrogen treatment (4).

To our knowledge, only eight cases of MESTK (four in girls and four in boys) have been described in children in scientific literature published in English (6–12). The pathogenesis of pediatric MESTK is not clear thus far (12). Owing to its rarity, only limited radiological information has been reported in the literature. The condition is commonly misdiagnosed as a malignant renal tumor by radiologists with insufficient understanding of MESTK. Here, we report a rare case of MESTK diagnosed in a 13-year-old girl and, for the first time, provide both CT and MRI findings of this rare tumor in children, along with a review of relevant literature.

## Case presentation

A 13-year-old girl presented to a local hospital with cough, nausea, and vomit persisting for over 1 month. Abdominal ultrasound revealed the presence of a mixed-echoic mass in the abdomen. Following this, she visited our hospital for further investigation. Upon physical examination, a 15 × 15 cm hard mass with poor mobility and without tenderness, rebound pain, or muscle tension was palpated in the abdomen. Laboratory examinations revealed that the level of red blood cells in the urine was elevated (167.8/µl; normal range, <23.0/µl). The level of cancer antigen 199 (CA199) was also slightly elevated (46.36 U/ml; normal range,<43.0 U/ml). The levels of tumor markers, namely alpha fetoprotein (AFP), carcinoembryonic antigen (CEA), and cancer antigen 125 (CA125), and hormones, namely estradiol (E2), progesterone (Prog), testosterone (Testo), luteinizing hormone (LH), follicle stimulating hormone (FSH), prolactin (PRL), and human chorionic gonadotropin (HCG), were all within normal limits.

**Figure 1:**
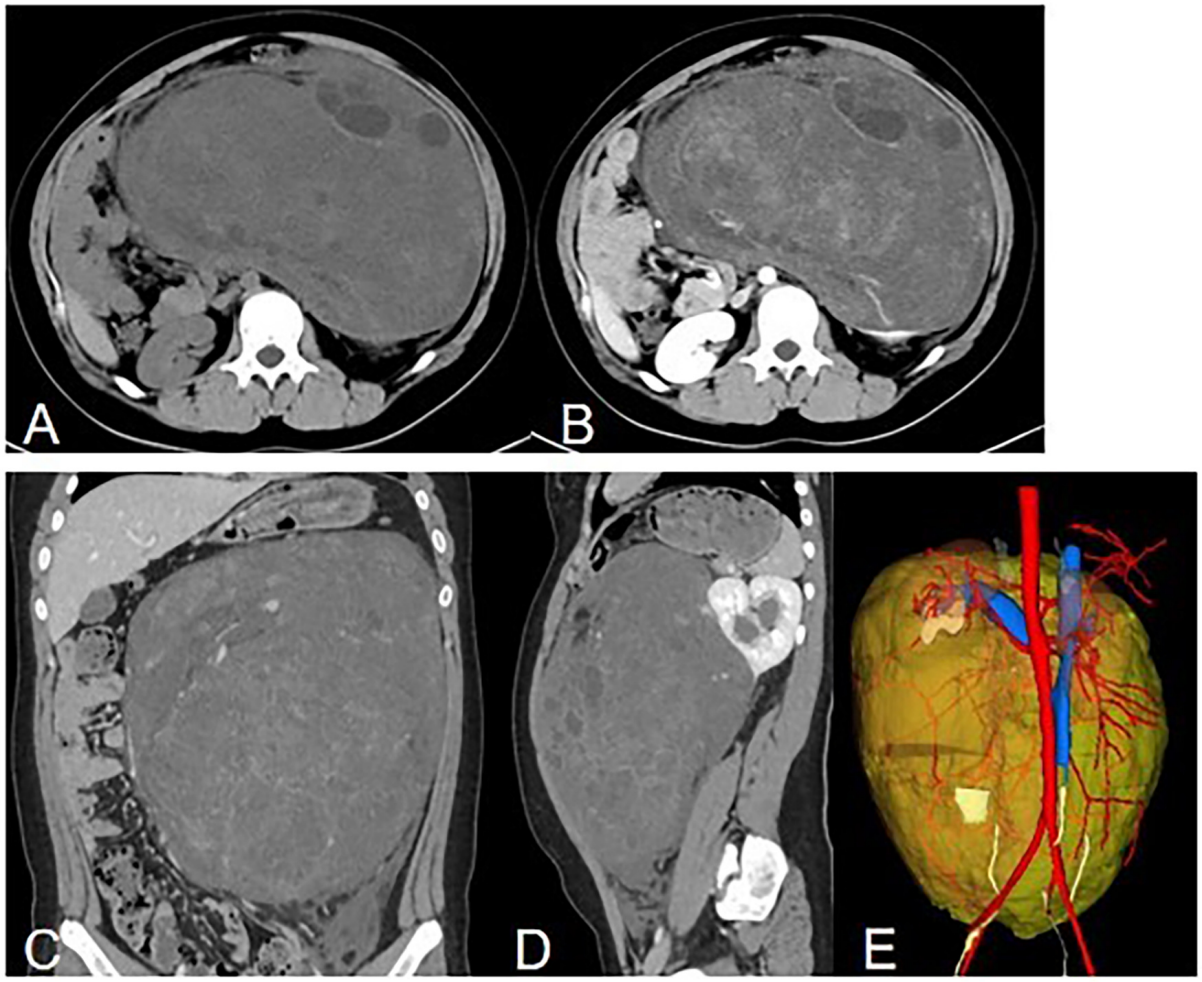
Computed tomography. **(A)** Non-enhanced axial image showing a huge, well-circumscribed, heterogeneous solid mass. **(B)** Axial delayed phase image showing persistent enhancement of the solid component and no enhancement of the cystic component. **(C)** Coronal image showing a heterogeneous enhanced mass. **(D)** Sagital image showing a heterogeneous enhanced mass arising from the left kidney with hydronephrosis. **(E)** 3D reconstruction of CT images. Posterior view is showed. The tumor is labeled with yellow, the left renal artery is labeled with rea and the left renal vein is labeled with blue.

**Figure 2:**
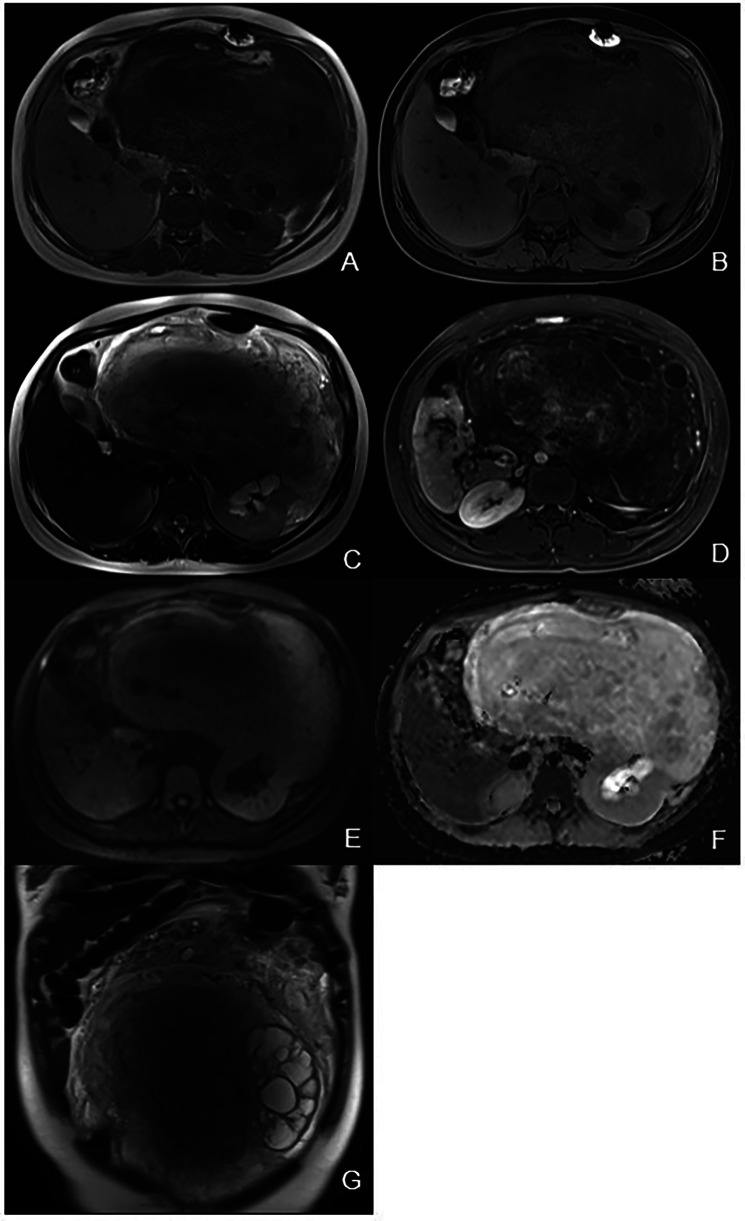
Magnetic resonance imaging. **(A)** Axial T1WI showing a heterogeneous hypo-intensity mass of the left kidney with area of high signal intensity. **(B)** Axial fat-suppression T1WI showing area of low signal intensity corresponding to adipose tissue. **(C)** Axial T2WI showing a heterogeneous hyper-intensity mass of the left kidney with hydronephrosis. **(D)** Axial delayed phase image showing a heterogeneous mass with cystic lesions. **(E)** DWI showing hyper-intensity of the mass. **(F)** ADC map showing hyper-intensity of the mass indicating no diffusion restriction. **(G)** Coronal T2WI showing a heterogeneous mass with multiple cystic lesions.

Abdominal contrast-enhanced CT (Figure 1) showed the presence of a heterogeneous solid mass with multiple cystic lesions arising from the lower pole of the left kidney, approximately 25.2 cm × 22.5 cm in size. In the arterial phase, the tumor showed mild enhancement and was supplied by the branch of the left renal artery. In the venous phase, the tumor showed persistent mild enhancement and drained into the left renal vein. In the delayed phase, the tumor showed mild enhancement. The large tumor involved the renal cortex, medullary, and calyces. This resulted in hydronephrosis and compressed the surrounding intestine and pancreas. Lymphadenopathy was not observed in the peritoneal and retroperitoneal space. MRI showed that the tumor had slight hypo-intensity on T1WI and hyper-intensity on T2WI with multiple cystic lesions, areas containing adipose tissue, and no diffusion restriction (Figure 2). The tumor showed mild enhancement in the arterial phase, persistent mild enhancement in the venous phase, and moderate enhancement in the delayed phase. The cystic lesions and areas with adipose tissues did not exhibit enhancement.

**Figure 3:**
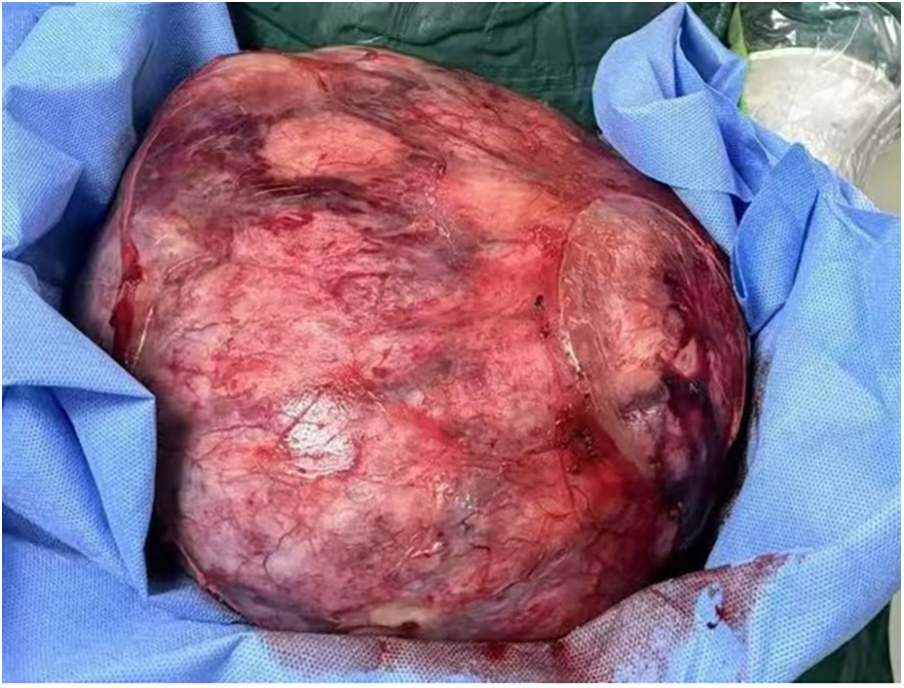
Gross specimen showing a well-defined mass measuring about 30 cm in its largest diameter with intact capsule.

The patient underwent a nephron-sparing surgery in the left kidney. Intraoperatively, a well-circumscribed, encapsulated, and tough tumor was observed in the middle and lower part of the left kidney. Its largest diameter measured approximately 30 cm, and it weighed approximately 5 kg (Figure 3).

**Figure 4:**
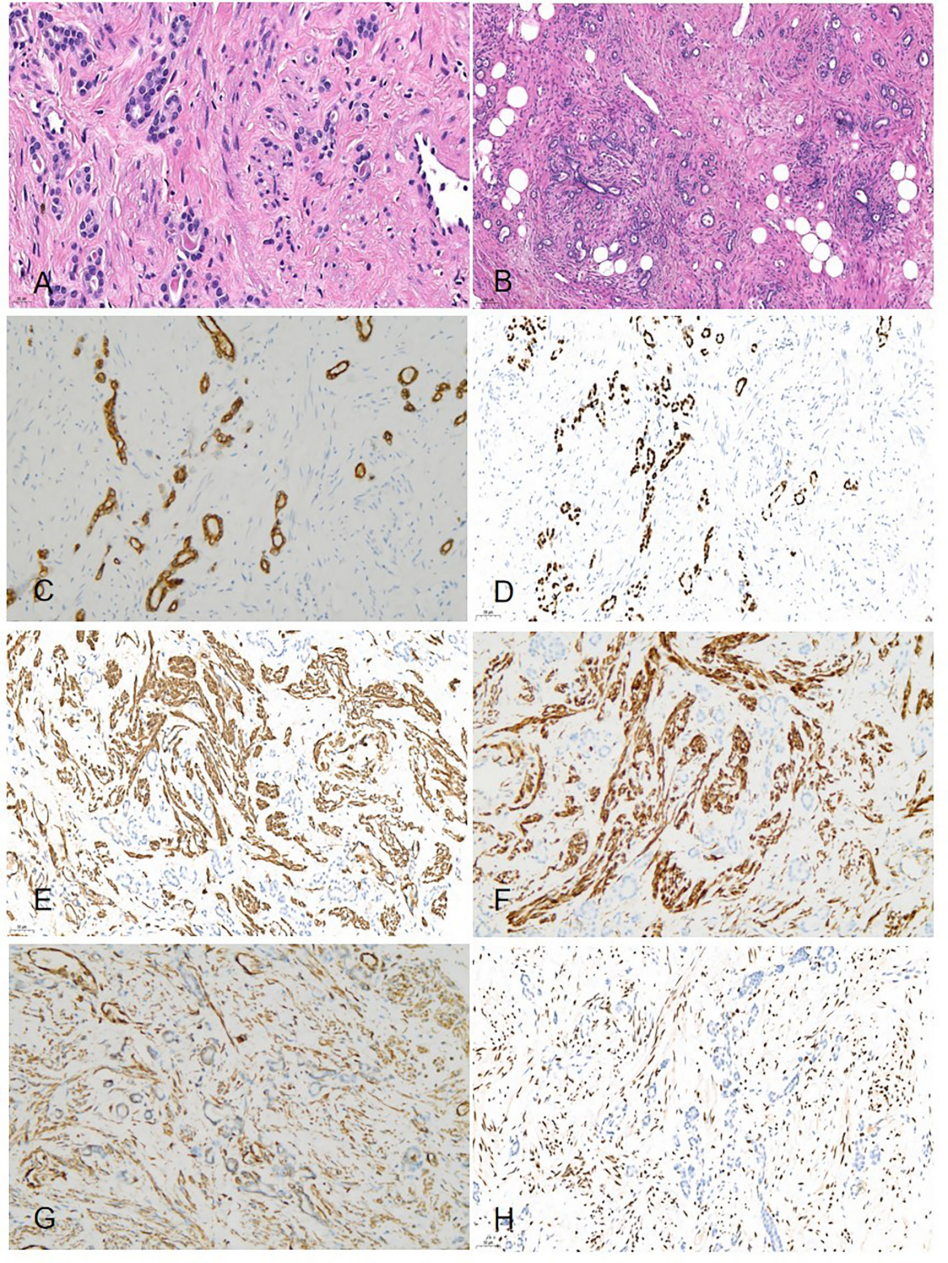
Histopathology **(A)** microscopically, the tumor was composed of a mixture of tubular architectures and hypocellular stromal fascicles (HE × 400). **(B)** Epithelial components that comprised tubules and cysts were scattered among stromal fascicles involving non-specific spindle cells (HE × 100). **(C)** Immunohistochemical studies showed the tumor cells were positive for CK-7 (IHC × 200). **(D)** The tumor cells were positive for PAX-8 (IHC × 200). **(E)** The tumor cells were positive for SMA (IHC × 200). **(F)** The tumor cells were positive for DES(IHC × 200). **(G)** The tumor cells were positive for vimentin(IHC × 200). **(H)** The tumor cells were positive for ER (IHC × 200).

Histologically, the border of the tumor was clearly delineated from the adjacent normal tissue. Owing to the large size of the tumor, we were unable to determine the origin from either the deep renal medulla or the peripelvic region of the kidney. The tumor was composed of tubular architectures and hypocellular stromal fascicles. These tubules were lined by columnar or hobnail epithelia, which were comparable to renal tubules or collecting ducts; however, immature epithelial elements typical of nephroblastoma were absent. These tubules exhibited no significant cellular atypia or necrosis, and we did not observe any mitotic figure. Cystic structures, varying in size (up to 10 mm), were also lined by stratified or occasionally by eosinophilic hobnail cells; however, urothelial differentiations were not observed. The stromal components were primarily composed of fibers, smooth muscles cells, and adipose tissue, which surrounded the tubular epithelial components. We observed neither mitotic features nor atypical cells (Figure 4). Findings from immunohistochemical studies showed that the epithelial component expressed CK-P, CK7, PAX-8, and EMA. The stromal cells tested positive for desmin, vimentin, smooth muscle actin (SMA), estrogen and progesterone receptors, and CD10 (Figure 4). The Ki-67 labeling index was 2%. Additionally, the tumor exhibited focal positive staining for CD34 and P504S. Eventually, the diagnosis of MESTK was established.

No adjuvant therapies were administered after surgery. The patient was free of disease during the follow-up period of over 1 year.

## Discussion

MESTK is a rare biphasic benign lesion that mostly originates from the müllerian tract. It predominantly occurs in perimenopausal women, and also in some adult males with long-term estrogen replacement; however, it rarely occurs in children (6). To date, only eight pediatric cases of MESTK have been described in scientific literature published in English, in four of which the children were prepubescent (8–12-year old), two were in preschool (3- and 4-year old), and two were pubescent males (14-year old) (6–12). Our patient was a 13-year-old girl, and this was the ninth known case of MESTK in children. As shown in Table 1, five girls and four boys have been diagnosed with this disease, including our patient. There were no obvious gender-based differences in prevalence among children.

**Table 1:** Clinical and MRI characteristics of patients with MESTK.

Reference	Age/sex	Symptoms	Size (cm)	Surgery	Outcome	MRI features
Hara et al. (6)	12/F	Abdominal mass	14	Radical nephrectomy	No recurrence (40months)	N/A
Goszczyk et al. (7)	12/F	Pyuria	N/A	Nefrectomy	N/A	N/A
Teklali et al. (8)	12/M	Hematuria	5	Partial nephrectomy	No recurrence (48months)	Hypointense on T1WI
Vergine et al. (9)	8/F	Abdominal pain	14	Nephrouretherotomy	No recurrence (16months)	N/A
Choy et al. (10)	14/M	Hematuria	2	Robotic partial nephrectomy	No recurrence (9months)	N/A
Elena et al. (11)	14/M	Blunt trauma hematuria	7	Robotic radical nephrectomy	No recurrence (18months)	N/A
Wei et al. (12)	3/F	Hematuria	10.2	Radical nephrectomy	No recurrence (3–6months)	N/A
Wei et al. (12)	4/M	Abdominal mass	16.9	Nephron sparing surgery	No recurrence (3–6months)	N/A
Present case	13/F	Cough, nausea and vomit	30	Nephron sparing surgery	No recurrence (12months)	Hypointense on T1WI and hyperintense on T2WI with multiple cystic lesions, areas containing adipose tissue, and no diffusion restriction
Adult	40–50/F	Abdominal mass, abdominal pain, hematuria, or asymptomatic	N/A	Partial or radical nephrectomy	N/A	High or equal intensity on T1WI and Hypointense on T2WI, cystic lesions, hemorrhage, calcification, and fat

F, Female; M, Male; N/A, Not available; T1WI, T1-weighted images; T2WI, T2-weighted images.

The primary clinical manifestations of MESTK include hematuria, abdominal pain, and the presence of an abdominal mass; however, some patients with MESTK remain asymptomatic (4, 8, 12, 13). As shown in Table 1, the clinical manifestations in children were consistent with those in adults. Our patient presented with cough, nausea, and vomit, which were different from the observations in previous pediatric cases. In our patient, the tumor was approximately 30 cm in size, which may have resulted in the compression of the surrounding intestines and subsequently led to nausea and vomit. As shown in Table 1, the tumor size ranged from approximately 2–16.9 cm in previous pediatric cases. The tumor dimension we reported was the largest among tumor dimensions reported in pediatric cases thus far. Additionally, the level of red blood cells in the urine were elevated in this case, which may be attributed to the invasion of the calyces by the tumor.

Histopathologically, most MESTKs comprise a stromal component (spindle cells) and epithelial component (cysts and tubules) (4, 13). These epithelial cells were arranged into various structures, including large cysts, small cysts, multiple cysts, microcysts, tubular structures, and papillae (13). However, Caliò et al. (4) reported that the hypocellular fibrous stroma and adipose tissue were more common in larger tumors. In our patient, the stroma was composed of hypocellular fibrous stroma and adipose tissue, which was compatible with the findings in the report. Immunohistochemical staining revealed that the epithelial component stained positive for CK, and the stromal component stained positive for vimentin, desmin, SMA, ER, and PR (14). Feng et al. (13) reported that both ER and PR were expressed in female patients, whereas they were not expressed in male patients. In our patient, we observed the expression of both ER and PR, which was consistent with the findings in the report.

The pathogenesis of adult MESTK is related to long-term estrogen replacement, long-term sex-steroid exposure, and high levels of interstitial sex hormone receptor expression (5). The pathogenesis of pediatric MESTK is currently unknown (10). Our patient was a 13-year-old adolescent girl with a 2-year history of menstruation. We speculated that the pathogenesis of her tumor may be related to sex hormone secretion disorder after initial menstruation. However, the precise pathogenesis needs to be investigated in depth.

The primary imaging modalities of MESTK include ultrasound, CT, and MRI. On ultrasound images, the tumor usually appears as a heterogeneous echoic mass, but it lacks specificity required for diagnosis. CT is a reliable diagnostic imaging tool for MESTK. On CT, MESTK presents as a heterogeneous, well-circumscribed, multiseptated cystic, solid-cystic or solid mass with delayed enhancement (Table 2) (15–19). In our case, the CT findings revealed the presence of a large heterogeneous mass with multiple cysts and mild enhancement in the solid component of the tumor. This was different from previous reports, as tumors of such large dimensions had not been reported before.

**Table 2:** Imaging features of patients with MESTK.

Reference	location	Exophytic	Imaging modality	Fat component	Characteristic	Boundary	Capsule	Enhancement degree
Hara et al. (6)	Right kidney	Yes	CT	No	Solid-cystic	Welll-circumscribed	No	Mild
Goszczyk et al. (7)	Right kidney	N/A	US, CT, MRI	N/A	N/A	N/A	N/A	N/A
Teklali et al. (8)	Left kidney	No	US, CT,MRI	No	Multicystic	Welll-circumscribed	No	N/A
Vergine et al. (9)	Right kidney	Yes	US, CT	No	Solid-cystic	Welll-circumscribed	Yes	Severe
Choy et al. (10)	Right kidney	No	US, CT	No	Multicystic	Welll-circumscribed	No	Mild
Elena et al. (11)	Right kidney	Yes	CT	No	Solid-cystic	Welll-circumscribed	Yes	Mild
Wei et al. (12)	Left kidney	Yes	CT	No	Solid	Welll-circumscribed	No	Moderate
Wei et al. (12)	Left kidney	Yes	CT	No	Soild	Welll-circumscribed	Yes	N/A
Present case	Left kidney	Yes	US, CT, MRI	Yes	Soild	Welll-circumscribed	Yes	Mild

US, Ultrasound; CT, Computed tomography; MRI, Magnetic resonance imaging; N/A, Not available.

The MRI findings of MESTK have different manifestations at different ages. Sahni et al. (16) and Domae et al. (17) reported that, in adult women, the solid component was hyperintense on T1WI and hypointense on T2WI, and the cystic component was hypointense on T1WI and hyperintense on T2WI. Ye et al. (20) and Kalinowski et al. (3) reported that the solid component was hypointense on T1WI and hyperintense on T2WI in two 18-year-old males. The MRI findings of our case showed that the tumor was hypointense on T1WI and hyperintense on T2WI, which was different from the MRI findings in adult women but similar to those in boys. MRI results showed multiple cystic lesions and areas containing adipose tissue in the tumor, characteristics that were consistent with the pathological findings of cystic structures and adipose tissue in the tumor. An important finding from the MRI results was that the tumor had no diffusion restriction, which suggested the benign nature of this tumor despite its large size. Additionally, the tumor showed mild to moderate enhancement on MRI, which may attributed to the hypocellular stromal components present in the tumor.

The major differential diagnosis of MESTK includes congenital mesoderm nephroma (CMN), cystic nephroma (CN), and nephroblastoma (Table 3).

1) CMN: CMN is a rare renal tumor that mostly occurs in infants and children, most of whom are less than 6 months in age (21). On CT, CMN shows solid or solid-cystic lesions with delayed enhancement. The solid components show moderate heterogeneous enhancement in the corticomedullary phase. The peripheral enhancement surrounding the tumor can manifest as a typical ring pattern and “double-layer sign” in the nephrographic phase, and the contrast agent excreted in the residual renal pelvis can manifest as the “intratumor pelvis sign” in the delayed phase (22). On MRI, CMN exhibits low intensity on T1WI, high, equal or low intensity on T2WI, and hyperintensity on DWI (22). Our patient was a 13-year-old girl. Her age of disease onset was different from that usually observed in CMN. Additionally, the tumor was a solid mass with multiple cystic lesions and exhibited mild delayed enhancement on CT. The feature of delayed enhancement was similar to that observed in CMN, but the typical “double-layer sign” and “intratumor pelvis sign” were absent.
2) CN: CN is a benign cystic kidney lesion that is more common in children aged between 3 months and 2 years and perimenopausal women. It constitutes 2.4% of primary renal tumors (19, 23). On CT, it appears as a non-reinforced cystic lesion with multiple loculations, and it is characterized by herniation into the renal sinus or renal pelvis. On MRI, it is hypointense on T1WI and hyperintense on T2WI (24). In our patient, the age of disease onset was different from that typically observed in patients with CN. The tumor was a heterogeneous solid mass, distinct from the cystic lesion usually observed in CN.
3) Nephroblastoma (Wilms tumor): Nephroblastoma mostly occurs in children, with a peak incidence age of 3–4 years. It accounts for 6% of all pediatric malignancies (19, 25). On CT, it may appear as a heterogenous enhanced solid mass with calcifications, fat, and vascular invasion (19, 25). On MRI, it exhibits low intensity on T1WI, variable or high intensity on T2WI, and restricted diffusion on DWI (19, 25, 26). Our patient's age of disease onset was different from that mostly observed in cases of nephroblastoma. Additionally, in our patient, the tumor exhibited no diffusion restriction, and the benign feature could be differentiated from the diffusion restriction of nephroblastoma.

**Table 3:** The major differential diagnosis of pediatric MESTK.

Differential diagnosis	Peak age	Symptoms	Size (cm)	Characteristic	CT features	MRI features
Congenital mesoderm nephroma	Less than 6 months	Abdominal mass, hypertension, or hematuria	3.3–20	Soild or solid-cystic	“Double-layer sign” “Intratumor pelvis sign”	Hypointense on T1WI, high, equal or low intensity on T2WI and hyperintensity on DWI
Cystic nephroma	3 months to 2 years	Abdominal mass	N/A	Cystic	Non-reinforced cystic lesion	Hypointense on T1WI Hyperintense on T2WI
Nephroblastoma	3–4 years	Abdominal mass	N/A	Soild	Heterogenous enhanced solid mass	Hypointense on T1WI, variable or high intensity on T2WI and restricted diffusion on DWI
MESTK	8–12 years	Abdominal mass, abdominal pain, hematuria, proteinuria or gastrointestinal symptoms	2–30	Soild, solid-cystic or multicystic	Heterogenous mass delayed enhancement	Hypointense on T1WI and hyperintense on T2WI with multiple cystic lesions, areas containing adipose tissue, and no diffusion restriction

CT, Computed tomography; MRI, Magnetic resonance imaging; T1WI, T1-weighted images; T2WI, T2-weighted images

Even though MESTKs are typically benign, rare malignant cases have been reported in adult patients (19, 27). Therefore, surveillance for metastatic spread or recurrence in these patients is necessary. All reported pediatric MESTKs were cured by surgical treatment and showed no recurrence during follow-up. Our patient was also disease-free during the follow-up over 1 year.

## Conclusion

In conclusion, MESTK is a rare biphasic benign nephroma in children. Findings from our report provide novel insights into the MRI features of a large pediatric MESTK and indicate the importance of MRI for observing adipose components and the absence of diffusion restriction within the tumor. Radiologists should consider the possibility of MESTK in children when they observe a large, solid renal tumor without diffusion restriction in children.

## Data Availability

The original contributions presented in the study are included in the article/Supplementary Material, further inquiries can be directed to the corresponding author.
